# The complete chloroplast genome of *Annona muricata* L.: a tropical fruit with important medicinal properties

**DOI:** 10.1080/23802359.2020.1820394

**Published:** 2020-09-16

**Authors:** Ying-Feng Niu, Kai-Xiong Li, Jin Liu

**Affiliations:** Yunnan Institute of Tropical Crops, Xishuangbanna, China

**Keywords:** *Annona muricata* L, chloroplast genome, sequencing

## Abstract

*Annona muricata* L. (*A. muricata*) is an important tropical fruit and medicinal plant. It is one of the easily found plants used traditionally in treating cancer. In many tropical countries, especially in Southeast Asia, *A. muricata* is popular for its edible fruit and medicinal merits. In this study, the complete chloroplast genome of *A. muricata* was sequenced, assembled, and annotated. The chloroplast genome of *A. muricata* was found to be a double strand ring structure with the size of 196,038 bp that consists of four regions: a large single-copy region of 75,339 bp, a small single-copy region of 3105 bp, and two inverted repeat regions of 58,797 bp. The GC content of the whole chloroplast genome was 39.92%. It was found that 111 protein-coding genes, one Pseudogene, 38 tRNA genes, and eight rRNA genes were annotated in the chloroplast genome, and the total number of genes was 158. DNA sequences of the chloroplast genomes of 19 species which belonged to three families of Magnoliales order were analyzed and a phylogenetic tree was constructed. The result indicated that *A. muricata*, *Annona cherimola*, *Uvaria macrophylla*, *Greenwayodendron suaveolens,* and *Chieniodendron hainanense* had a close phylogenetic relationship. The findings also provided abundant basic data for the genomics study of *A. muricata.*

*Annona muricata* L. is an important tropical fruits that contributes to the economic growth of some tropical countries that include tropical America, Australia, Africa and Malaysia (Cheong et al. 2011). It belongs to the family Annonaceae, which is commonly known as Soursop, Graviola and Guanabana (Soheil et al. 2015). In addition to being a fruit tree, *A. muricata* also has medicinal properties and has been traditionally used to treat cancer (Minari and Okeke 2014). The compounds isolated from the seeds and the leaves of *A. muricata* showed significant *in vitro* cytotoxicity toward the human hepatoma cell lines (Liaw et al. 2002; Chang et al. 2003). Besides, the leaf decoction is usually administered to lessen the symptoms of cancer (Mao et al. 2011). *A. muricata* also has many other medicinal effects. The phytochemical complexity of *A. muricata* extracts may offer health-promoting benefits including chemotherapeutic and chemopreventive effects (Yang et al. 2015). Animal experiments have stated that the ethanol extract from the stem bark of *A. muricata* significantly inhibited cold immobilization stress-induced increase in lipid peroxidation in the liver and brain of albino rats (Padma et al. 1997). The aqueous extract has been found to protect the pancreatic β-cells against streptozotocin-induced diabetic rats (Florence et al. 2014).

In many tropical countries, especially in Southeast Asia, *A. muricata* is popular for its edible fruit and medicinal benefits (Ragasa et al. 2012; Ana et al. 2016). Until now, there have been many studies on *A. muricata*, but most of them are about its medicinal effect or chemical compounds obtained from it. However, there are very few reports on the genomics of *A. muricata*, and the chloroplast genome has not been reported yet. In this study, the chloroplast genome of *A. muricata* was sequenced, assembled, and annotated.

Fresh tender leaves of *A. muricata* were collected from the Xishuangbanna Tropical Flowers and Plants Garden (100.70421 E, 22.015894 N) and the genomic DNA was isolated using the Dneasy Plant Mini Kit (Qiagen). The purified DNA was stored in the ultra-low temperature specimen library at YITC (specimen accession number: YITC-2020-FZ-A-003). The sequencing library was constructed and DNA sequencing was carried out by the Illumina Hiseq 2500 Platform (Illumina, San Diego, CA). Chloroplast genome assembly and annotation was carried out by CLC Genomics Workbench v3.6 (http://www.clcbio.com) and DOGMA (Wyman et al. 2004). The assembly and annotation results of the chloroplast genome of *A. muricata* were uploaded to the GenBank (http://www.ncbi.nlm.nih.gov/) under the accession number MT742546.

The same as other plants, the chloroplast genome of *A. muricata* was found to be double stranded ring structure with the size of 196,038 bp and consist of four regions: a large single-copy region (LSC, 75,339 bp), a small single-copy region (SSC, 3,105 bp), and two inverted repeat regions (IRa and IRb, 58,797 bp). Compared to the chloroplast genomes of other plants, the *A. muricata*'s IR region was found to be large and the SSC region was small. The GC contents of LSC, SSC and IR regions of the chloroplast genome were 38.84%, 45.07% and 40.73%, respectively. The GC content of the whole chloroplast genome was 39.92%. The whole chloroplast genome composed of 59,091 A bases (30.14%), 58,697 T bases (29.94%), 39,673 G bases (20.24%), and 38,577 C bases (19.68%). There were 111 protein-coding genes (PCGs), one Pseudogene, 38 tRNA genes, and eight rRNA genes that were annotated in the chloroplast genome of *A. muricata*. The total number of genes was 158. Among the 111 protein-coding genes, *ycf2* was the largest gene with the size of 7,518 bp and *petN* gene was the smallest with the size of 90 bp.

In order to study the phylogenetic relationship between *A. muricata* and other plant species, 19 plant species were considered which were closely related to *A. muricata*, and a maximum likelihood tree based on the DNA sequences of the protein-coding genes common to chloroplast genomes of all species was constructed (Figure 1). The 19 species belong to three families of Magnoliales order, of which 13 species belong to the Magnoliaceae family, 5 species belong to the Annonaceae family and only one species belongs to the Myristicaceae family. *Kadsura longipedunculata* belong to Schisandraceae family of Austrobaileyales order which was used as the out-group. The multiple sequence alignment was carried out by MAFFT (Katoh and Standley 2013) and maximum-likelihood (ML) analysis was carried out by MEGA7.0 (Kumar et al. 2016). In the phylogenetic analysis, 20 species were divided into three groups, among which *A. muricata* and *Annona cherimola*, *Uvaria macrophylla*, *Greenwayodendron suaveolens,* and *Chieniodendron hainanense* were added in the same group. The results indicated that they had a close phylogenetic relationship. This study not only reported the complete chloroplast genome of *A. muricata* firstly but also provided abundant basic data for the genomics study of *A. muricata*.

**Figure 1.**
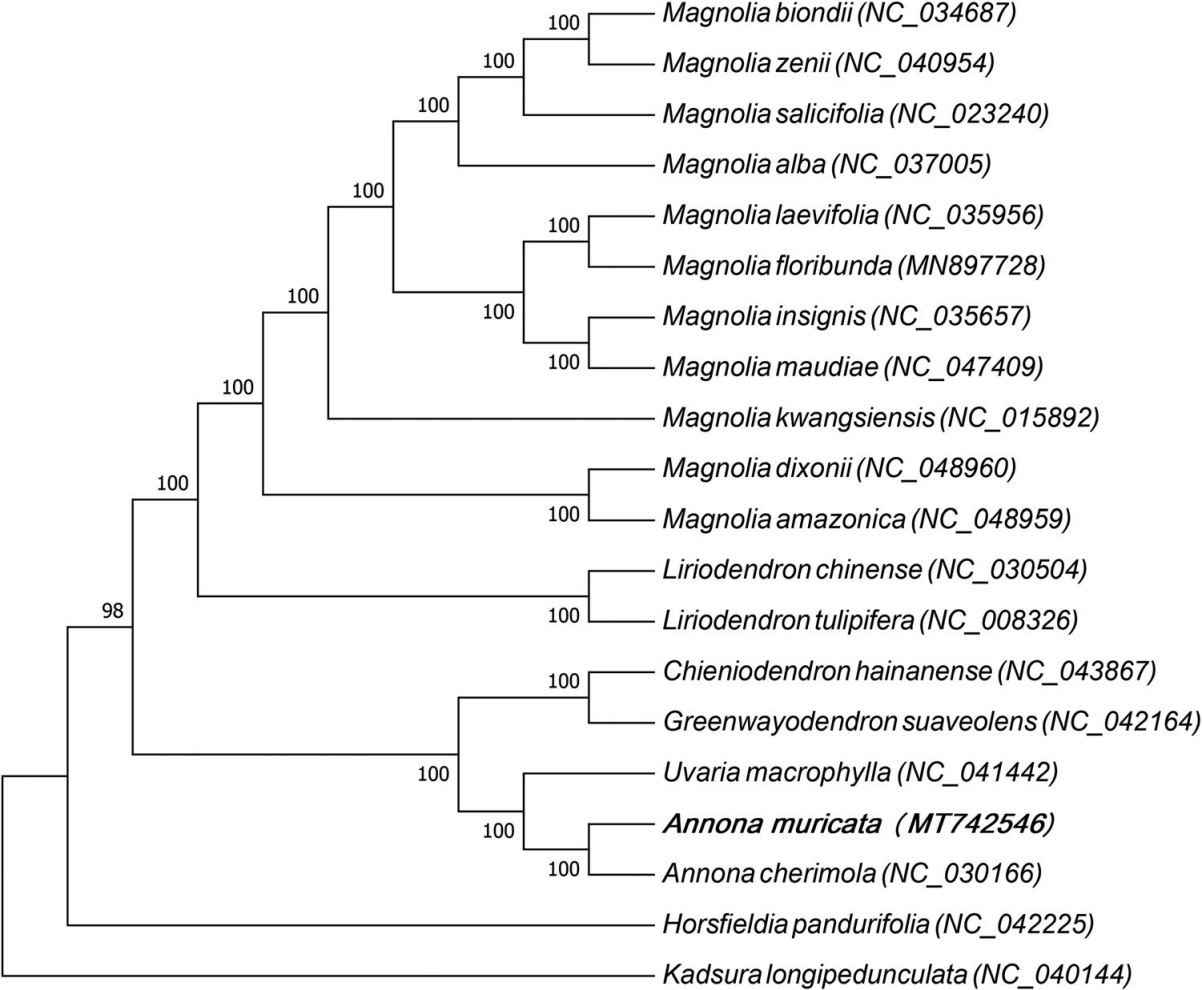
Maximum likelihood tree based on the sequences of protein coding genes of 20 plant species, 13 species belong to Magnoliaceae family, 5 species belong to Annonaceae family and only one species belongs to Myristicaceae family, and *Kadsura longipedunculata* which belongs to Schisandraceae family of Austrobaileyales order was used as the out group.The species and chloroplast genome accession numbers for the phylogenetic tree construction are: *Magnolia biondii* (NC_034687)*, Magnolia zenii* (NC_040954), *Magnolia salicifolia* (NC_023240), *Magnolia alba* (NC_037005), *Magnolia laevifolia* (NC_035956), *Magnolia floribunda* (MN897728), *Magnolia insignis* (NC_035657), *Magnolia maudiae* (NC_047409), *Magnolia kwangsiensis* (NC_015892), *Magnolia dixonii* (NC_048960), *Magnolia amazonica* (NC_048959), *Liriodendron chinense* (NC_030504), *Liriodendron tulipifera* (NC_008326), *Chieniodendron hainanense* (NC_043867), *Greenwayodendron suaveolens* (NC_042164), *Uvaria macrophylla* (NC_041442), *Annona muricata* (MT742546), *Annona cherimola* (NC_030166), *Horsfieldia pandurifolia* (NC_042225), and *Kadsura longipedunculata* (NC_040144).

## Data Availability

The chloroplast genome data that support the findings of this study are openly available in GenBank at https://www.ncbi.nlm.nih.gov/, reference number MT742546. The raw sequencing data are openly available in SRA database with the accession number PRJNA658436 and SRR12506317.
